# Temporal patterns in taste sensitivity

**DOI:** 10.1093/nutrit/nuad097

**Published:** 2023-08-09

**Authors:** Andrew Costanzo

**Affiliations:** CASS Food Research Centre, School of Exercise and Nutrition Sciences, Deakin University, Geelong, Victoria, Australia

**Keywords:** circadian, diurnal, gustation, menstrual, seasonal, sensitivity, taste

## Abstract

Individuals vary in their ability to taste, and some individuals are more sensitive to certain tastes than others. Taste sensitivity is a predictor of various factors, such as diet, eating behavior, appetite regulation, and overall health. Furthermore, taste sensitivity can fluctuate within an individual over short to long periods of time: for example, in daily (diurnal) cycles, monthly (menstrual) cycles (in females), and yearly (seasonal) cycles. Understanding these temporal patterns is important for understanding individual eating habits and food preferences, particularly in the context of personalized and precision nutrition. This review provides a summary of the literature on taste sensitivity patterns across 3 temporal dimensions: daily, monthly, and yearly. Good evidence for diurnal patterns has been observed for sweet taste and fat taste, although the evidence is limited to rodent studies for the latter. Obese populations showed limited variation to sweet and fat taste sensitivities over a day, with limited variation in sweet taste sensitivity being linked to insulin resistance. There were mixed observations of temporal variation in sensitivity to sour and umami tastes, and there were no patterns in sensitivity to bitter taste. Menstrual patterns in sweet taste sensitivity were consistent with patterns in food intake. Other taste modality investigations had mixed findings that had little agreement across studies. Hormonal changes in females influence taste sensitivity to some degree, although the overall patterns are unclear. Seasonal patterns have been less well studied, but there is weak evidence that sweet, salty, and bitter taste sensitivities change across seasons. Differences in seasonal taste patterns have been observed in subgroups susceptible to mental health disorders, requiring further investigation. Patterns of taste sensitivity are evident across multiple temporal dimensions, and more research is needed to determine the influence of these patterns on food intake. Dysregulation of these patterns may also be a marker of certain diseases or health conditions, warranting further investigation. Notably, the alimentary tastes (umami, fat, and carbohydrate) are underrepresented in this research area and require additional investigation.

## INTRODUCTION

Taste sensitivity, which is the ability to perceive or identify a taste stimulus, has a clear impact on an individual’s food intake and eating behavior. In general, people who are more sensitive to certain tastes tend to consume less of foods that exhibit that taste, while individuals with lower sensitivity to certain foods require stronger taste sensations or greater food intake to experience the same level of perception.^1^ For example, individuals with a heightened sensitivity to bitter tastes may avoid certain vegetables that have a bitter taste, whereas those with lower sensitivity may consume these vegetables more easily.^2–3^ Additionally, individuals with lower taste sensitivity may be more likely to consume unhealthy foods, such as those high in sugar or salt,^4^ eating behavior associated with the onset of obesity, diabetes, and other health problems.^1^ The magnitude of differences in the ability to detect certain tastes between individuals can range from 120- to 1000-fold in concentration.^5–7^ Personalized and precision nutrition that tailors dietary recommendations to individual taste sensitivities, among other factors, can help individuals make healthier food choices. By understanding individual taste sensitivities, it is possible to create a more personalized dietary plan that considers dietary preferences and appetitive signaling, and this may help those individuals to maintain a healthy diet. Therefore, understanding the relationship between taste sensitivity and food intake is crucial for promoting healthy eating habits and improving overall health.

The gustatory (or taste) system is, in short, the detection and perception of soluble chemicals (also known as tastants) in the oral cavity (ie, sucrose is a tastant for sweet taste). The primary role of the basic taste system is as a gating mechanism, in that healthful and nutritive tastants are accepted and ingested, while harmful or distasteful chemicals are rejected and expectorated.^8^ The taste modalities in this system include sweet, salty, sour, bitter, and (arguably) umami. Tastants from each taste modality are perceived in the oral cavity via the activation of taste receptors specific to each modality. The main sweet taste receptor is a dimer of 2 proteins, TAS1R2 and TAS1R3.^9^ Twenty-five bitter taste receptors in the TAS2R family have been found in human taste cells, each specific to particular bitter compounds.^9^ A sour taste receptor, OTOP1, has recently been identified.^10^ Research regarding the identification of a salty taste receptor is ongoing.^11^

Umami (or savory) taste may be considered a basic taste, but it has recently been suggested that it be recategorized into a new category termed “alimentary tastes,”^12^ along with other newly discovered taste modalities.^13^ The role of the alimentary tastes is post-ingestive, in that the alimentary taste sensations influence subsequent behavior throughout the eating event, particularly via satiety and satiation signaling. Basic tastes influence acute food choices in relation to the food that is currently being tasted, whereas alimentary tastes affect short-term behavior in relation to foods to be consumed subsequently. The alimentary tastes include umami,^14^ fat taste,^15^ and carbohydrate taste,^16^ and they enable the detection of amino acids, fatty acids, and complex carbohydrates, respectively. Since these tastes correspond to the 3 macronutrient types, they have an important and relevant influence on eating behavior and energy intake.^13^ The umami receptor is a dimer of TAS1R1 and TAS1R3, sharing 1 subunit with the sweet taste receptor.^9^ A range of fat taste receptors have been identified in humans,^17–18^ with CD36 and FFAR4 being implicated as the primary receptors involved in fatty acid chemoreception.^19^ Research on carbohydrate taste is relatively young, and there is ongoing research to study its role in regulating food intake and the receptors involved in complex carbohydrate chemoreception, but there is evidence suggesting that carbohydrate taste receptors differ from sweet taste receptors.^16^

**Figure 1:**
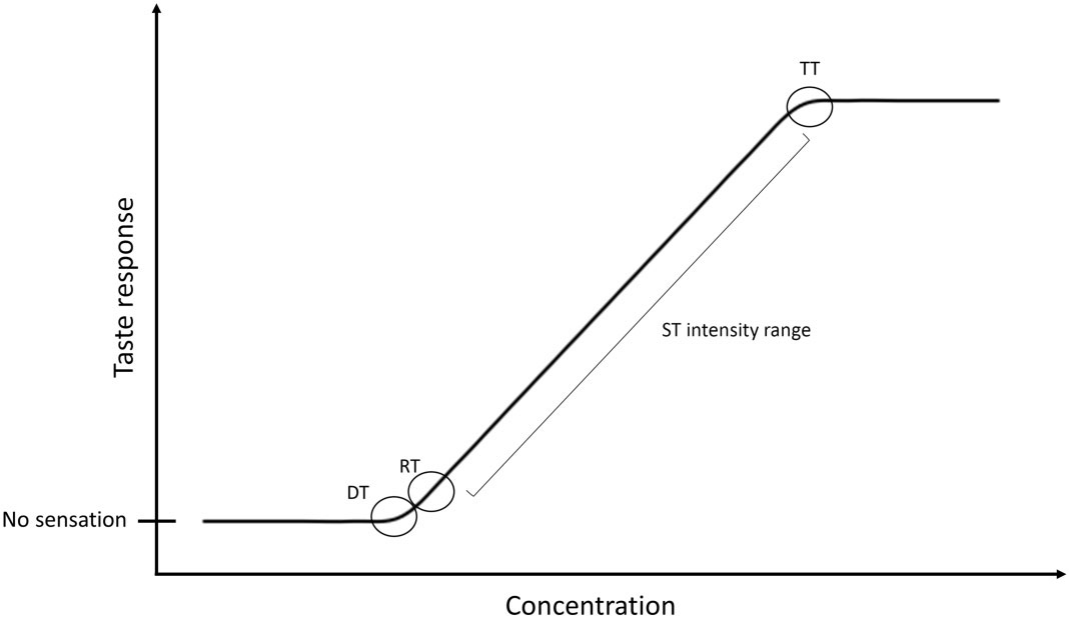
**Relationship between tastant concentration and perceptual taste responses.** *Abbreviations:* DT, detection threshold; RT, recognition threshold; ST, suprathreshold; TT, terminal threshold.

Traditionally, psychophysical methods are used to determine taste thresholds as a measure of taste sensitivity. The detection threshold (DT) is the minimum amount of a stimulus needed to provide a perceptual sensation of any kind; the recognition threshold (RT) is the minimum amount of a stimulus needed to provide a perceptual sensation from which the sensory descriptor is convincingly recognizable; and the terminal threshold is the minimum amount of a stimulus above which no difference in intensity can be perceived (Figure 1).^20^ The intensity of a perceived stimulus (recognized stimuli are registered at stimulus strengths between the RT and the terminal threshold, called the suprathreshold [ST] intensity range) is also used as a subjective measure of taste sensitivity.^20^ The dimensions of taste perception do not necessarily correlate with one another and represent different processes of gustation.^21^ Since the discovery of taste receptors,^22^ molecular analyses have been used as markers of taste function, especially in animal models. This generally takes a transcriptomic approach, in which receptor gene expression, or RNA, is measured as an indicator of receptor activity,^23^ but receptor proteins or metabolites are also sometimes assessed.^24^ For example, expression of respective receptor genes is associated with sweet,^25^ bitter,^26^ umami,^27^ and fat taste^28^ sensitivities in animal models. While this can lead to a more objective measure of the taste mechanisms within the oral cavity, it may involve more invasive or burdensome techniques, such as biopsies,^23^ and it does not consider neural signaling or central processing, which can each affect the final perceived sensation.^29^

In most taste research, taste thresholds are measured at a single time point and extrapolated to represent overall taste sensitivity for a given modality. However, taste sensitivity changes regularly, and there can be long-term (eg, month-to-month) to short-term (eg, hour-to-hour) variation. There is evidence to suggest that taste receptors expressed on a taste cell are regulated rapidly and dynamically throughout feeding or oral exposure to tastants,^24^ demonstrating the immediacy of changes in gustation in response to environmental cues. Cyclical patterns of taste sensitivity for multiple modalities have been observed across hours,^30–31^ weeks,^32–34^ and months.^35–36^ It is important to note that the patterns described here are not the same as the decline in taste sensitivity associated with aging, which is outside the scope of this article. Rather, the patterns being reviewed here present as rises and falls in taste sensitivity in a cyclic pattern.

Understanding these patterns can aid future nutrition research, as the temporal patterns may be linked to the timing of an individual’s food choices, cravings, snacking, and other nuances of eating behavior. From a public health perspective, insights into temporal patterns of taste sensitivity may aid in the formulation of food recommendations, such as optimal eating timing. From a personalized and precision nutrition perspective, understanding the predictors of food intake at any given time is crucial, and this includes variations in taste sensitivity. As explained earlier, how a person perceives nutritive compounds can affect their food choices, intake quantities, and health outcomes.^1^ The temporal patterns that are unique to an individual may be a predictor of food intake and should be a consideration in designing personalized nutrition plans. From a research methodology perspective, these temporal patterns should be considered in future nutrition and psychophysics studies. Many studies do not control for the time of day, month, or year in which data is collected, but these variations in time may influence the research outcomes.

## METHODS

Literature searches were conducted in reference databases such as PubMed, Scopus, and Embase in January 2023. The following keywords were used in multiple combinations: “taste”, “gustatory”, “threshold”, “temporal”, “pattern”, “rhythm”, “diurnal”, “circadian”, “daily”, “seasonal”, “annual”, “yearly”, “monthly”, and “menstrual”. Titles and abstracts were screened manually and shortlisted, if relevant. The full texts of the shortlisted articles were then reviewed manually and excluded if it was determined they were not relevant. The reference lists of the collected articles were then reviewed manually to identify any relevant articles that may have been missed in the literature searches.

## DAILY (DIURNAL AND CIRCADIAN) PATTERNS

There are several human studies that have demonstrated cyclical patterns in taste sensitivity across a day, with most studies observing changes in sensitivity occurring on the scale of 2 hours–3 hours.^30–31^^,^^37–39^ The mechanism for this change is likely related to rapid regulation of taste receptor expression on taste bud cells. Animal studies have shown that taste receptor expression on a taste bud cell can be downregulated immediately following oral exposure to relevant stimuli.^24^^,^^28^ Whether the changes to receptor expression are solely regulated by environmental stimuli such as food consumption, or if there are also endogenous internal clocks that control gustatory function within a day, is largely underresearched. It is probable that these 2 factors occur in tandem, and that, in part, they are responsible for taste-mediated appetite. For example, periods of fasting may lead to a gradual upregulation of taste receptor expression and sensitivity, resulting in a greater number of unoccupied receptors and a desire for foods containing compounds of a particular taste.^40^ Once foods have been ingested and taste receptors activated, receptor expression is downregulated rapidly to maintain homeostatic balance of energy intake. This phenomenon is known as sensory-specific satiety.^41^

Daily patterns of food intake are well researched.^42^ Typically, humans in Western society consume more energy in the evening (ie, at dinner) compared with the morning (ie, at breakfast).^43^ In addition, the types of nutrients consumed at different parts of the day may also vary, with more carbohydrates being consumed in the morning and more fats in the evening.^43^ This may be to satisfy the varying nutritional requirements throughout the day, such as the loading of energy-dense foods in the evenings in anticipation of an overnight fast.^44–45^ There are, however, a number of social and physiological factors that may drive these nutritional requirements and eating behaviors, and they go beyond the gustatory system.^42^ These factors are not explored in this review, but it should be noted that drivers of food intake are complex and multifactorial. Regarding the role of taste in regulating intake based on nutritional requirements, daily patterns of taste sensitivity may be relevant. In particular, patterns of specific alimentary taste sensitivities may be responsible for the intake patterns of carbohydrate, protein, and fat (eg, fat taste sensitivity may decrease in the evenings to promote greater fat intake). The interplay between the multiple taste modalities, each with differing daily patterns, may represent or explain eating behavior within a day, including the timing of meals and snacks.

Daily patterns or rhythms can be observed in 2 different ways, and it is important not to conflate the data for these, as they may be related to different patterns of taste sensitivity. Diurnal rhythms are linked to the daylight hours within a day (ie, the light hours vs the dark hours), whereas circadian rhythms are linked to the awake hours of the day (ie, hours awake vs hours asleep). For most individuals, these 2 rhythms overlap, but researchers should be mindful that this may not be the case in some population subgroups, such as night shift workers or people that live in extreme latitudes of the earth, where daylight hours may be limited or prolonged.

In this section, tastes are presented separately. No studies have been conducted on the daily patterns of carbohydrate taste sensitivity, so it is not presented in this review.

### Daily patterns in sweet taste sensitivity

Three studies have assessed daily patterns of sweet taste sensitivity in humans. The earliest study to do this assessed the sucrose RT in 16 participants in the United States at 4 times within a day: 1000 h, 1100 h, 1330 h, and 1630 h.^37^ Each participant repeated the study multiple times on different days, with 7–18 repetitions completed for each participant. Subjects were provided with an optional lunch between 1200 h and 1300 h. Higher RTs were observed at 1000 h and 1330 h, which occurred after breakfast intake and lunch intake, respectively, and lower RTs were observed at 1200 h and 1630 h, which were after periods of fasting. The researchers also compared changes in the RT between the days on which participants chose to or declined to eat lunch. In this subanalysis, it was shown that omitting lunch led to a gradual decrease in the RT throughout the day, with no peaks occurring. This suggests that recent meal intake increases the sweet taste RT (ie, decreases sweet taste sensitivity).

Two Japanese studies from the same research group assessed the RT of multiple sweet taste stimuli (sucrose, glucose, and saccharin) in healthy weight^30^ and overweight and obese^31^ participants, using similar methods. In both studies, RTs were measured 30 minutes prior to a meal, and 1 h after a meal, between 0800 h and 2200 h. Mealtimes occurred at 0830 h (breakfast), 1230 h (lunch), and 1730 (dinner). In the study assessing the healthy weight cohort,^30^ participants were recruited into 3 different meal protocols: 3 meals (breakfast, lunch, and dinner; n = 47), 2 meals (lunch and dinner; n = 16), or 1 meal (dinner; n = 24). Some participants repeated the study and overlapped between groups. In general, sucrose RTs increased throughout the day in all protocols, although the greatest increase was seen in the 3-meals group. Glucose RTs also increased in the 2- and 3-meals groups, but not in the 1-meal group. Saccharin RTs only increased in the 3-meals group. These findings suggest that more frequent meals lead to a greater rise in sweet taste RTs (decreased sweet sensitivity), which matches what was found in the earlier study.^37^ Differences in the results between the various sweet tastants may be due to different taste mechanisms. For example, recent evidence has suggested that chemoreception of glucose may occur via a sodium–glucose cotransporter pathway, separate from the TAS1R2 and TAS1R3 dimer.^46^ In the study assessing the overweight and obese cohort,^31^ only the 3-meals protocol was used (n = 36). Females only participated on days that were 3 days–16 days after the end of the previous menstrual phase, so the results would not be influenced by hormonal cycles. In addition to taste assessment, participants had blood samples drawn to assess their insulin and glucose levels, in order to model insulin resistance scores. No patterns were observed for the RTs for any of the 3 sweet taste stimuli used with this group. Interestingly, it was shown that insulin-resistance scores were negatively associated with percentage change in sweet taste RT across the day for all 3 sweet taste stimuli. The authors propose that those who are more insulin resistant exhibit diminished diurnal patterns in their sweet taste RT and are less able to regulate their sweet taste sensitivity in response to meal intake,^31^ which may be a marker for diabetes and other metabolic diseases.

A 2022 study compared sucrose DTs between Brazilian participants with different work/study schedules.^47^ Although the primary aim of this study was not to observe diurnal patterns in taste sensitivity, the results do provide some insights into the matter. Participants were categorized into 3 groups (n = 37 for each group): a control group (who studied or worked during regular daylight hours); students who attended school/classes late in the evening after normal working hours (group 1); and staff (eg, medical professionals) who work overnight shifts at a hospital (group 2). DTs were assessed between 0900 h and 1100 h or between 1400 h and 1600 h in the control group, and between 1800 h and 1900 h (prior to the class or shift) in groups 1 and 2. Groups 1 and 2 had higher DTs than the control group (mean DT ± SD: group 1 = 4.78 ± 3.12 g/L; group 2 = 4.24 ± 1.88 g/L; control group = 2.33 ± 1.84 g/L). It was difficult to differentiate between the effects of diurnal and circadian rhythms in this study, due to the study protocol, but it was interesting that there were differences in the DTs collected at different times of the day. These results also matched the earlier studies,^30–31^^,^^37^ in that thresholds were greater (sensitivity was lower) in the evening.

Two mice studies have been conducted on sweet taste receptor gene expression patterns within a day, with mixed findings. One study compared *TAS1R2* and *TAS1R3* expressions in a range of tissue, including tongue, at 0700 h and 1900 h.^48^ In lingual tissue, both receptor gene expressions were lower at 1900 h compared with 0700 h, although the differences were not statistically significant. Interestingly, these same receptors in stomach and gastrointestinal tissue demonstrated significant differences between time points, so perhaps sweet taste receptors are regulated locally within tissues rather than systemically. Another study assessed *TAS1R3* expression in taste papillae between lean and obese mice across multiple time points within 24 hours.^49^ These mice had free access to chow and water and tended to consume most of their food and energy during dark hours (from 2200 h). In lean mice, *TAS1R3* expression remained stable across most of the 24 hours, but there was a notable sharp increase in expression toward the end of daylight hours (1900 h), which was suggested to be in preparation for seeking food (increased hunger and desire to eat) once darkness has set. Conversely, the obese mice did not show significant variation or temporal patterns in *TAS1R3* expression across 24 hours. This matches the findings in humans,^30–31^ in which obese individuals had a diminished ability to regulate sweet taste sensitivity, compared with lean individuals.

In the 3 human studies that tracked sweet taste sensitivity over time,^30–31^^,^^37^ there did not appear to be consistent daily patterns in sweet taste sensitivity. Average threshold peaks and troughs occurred at different time points when comparing studies, and when comparing different individuals within studies. The only consistent finding was that food intake seemed to be main regulator of sweet taste sensitivity^30^^,^^37^ and receptor expressions.^48–49^ Markers of taste sensitivity or function increased in the lead up to food intake (ie, during periods of fasting) and decreased following meal intake, although this was only observed in healthy populations. In the obese humans^31^ and mice,^49^ thresholds were resistant to food intake and were related to insulin resistance. This is early evidence of a relationship between the ability to regulate sweet taste sensitivity and metabolic syndrome. Thus, sweet taste sensitivity patterns within an individual, as opposed to sweet taste sensitivity at a single time point, may be a potential marker of health status or predisposition to poor health.

### Daily patterns in salty taste sensitivity

Three studies have assessed daily patterns in salty taste sensitivity, all conducted within Japanese populations. In 2 studies (methods outlined in the Daily patterns in sweet taste sensitivity section), there were no observed patterns in salty taste RTs in healthy or obese individuals.^30–31^

In a separate study^39^, 6 young males were observed over 2.5 days, and their salty taste RTs were assessed at 0900 h, 1200 h, 1500 h, 1800 h, and 2100 h on day 2, and at 0000 h, 0300 h, 0600 h, and 0900 h on day 3. This is currently the only study to track taste thresholds overnight in humans. Two meals containing a moderate amount of sodium chloride (3 g) were provided to participants each day, between 1230 h and 1300 h, and between 1830 h and 1900 h. There was an overall pattern in salty taste sensitivity, with low RTs at 1500 h and high RTs at 0000 h, although it should be noted that unique temporal patterns were observed in individual participants. RTs did not appear to be associated with meal intakes, but the authors acknowledge that the participant sample or meal patterns in this study may not be representative of a free-living population.

The results are mixed on whether there are daily patterns in salty taste sensitivity, with only 1 of 3 studies reporting observed patterns.^39^ However, that study measured RTs across a full 24 hours rather than 14 hours and reported a significant peak in thresholds throughout the night, during typical sleeping hours. The magnitude of difference between daylight and dark hours may have driven the statistically significant finding, which may be regulated by internal clock mechanisms^48^ rather than food intake. Further studies with well controlled study protocols are needed.

### Daily patterns in sour taste sensitivity

Three studies have assessed daily patterns in sour taste sensitivity. In 2 Japanese studies (methods outlined in the Daily patterns in sweet taste sensitivity section), there were no observed patterns in citric acid RTs in healthy or obese individuals.^30–31^ Another study conducted in the United States assessed daily patterns in hydrochloric acid DTs under different protocols.^38^ In Group 1, 4 male participants attended 4 days of testing for each of the following protocols: usual lunch, no lunch, and high-energy lunch (12 days total). In Group 2, a separate sample of 4 male participants attended 4 days of testing for each of the following protocols: usual lunch, late lunch, and high-energy lunch (12 days total). DTs were measured approximately every 1 hour to 3 hours from 0800 h to 1600 h. In Group 1, during the “no lunch” protocol, DTs decreased throughout the day. Other than with that protocol, all groups and protocols showed increased DTs following lunch, regardless of the lunch timing or the energy content. The results from this study suggest that sour taste sensitivity is modulated by food intake, as thresholds were only decreased when lunch was omitted entirely.

There were mixed results for the daily patterns in sour taste sensitivity. There was some evidence for sour taste sensitivity being regulated by meal intake, although it is difficult to extrapolate this to a wider audience, due to the low sample size.^38^ More research is needed to determine the patterns in sour taste sensitivity.

### Daily patterns in bitter taste sensitivity

Two studies have assessed daily patterns in bitter taste sensitivity, both from the same research group in Japan (methods outlined in the Daily patterns in sweet taste sensitivity section).^30–31^ In both studies, there were no observed diurnal patterns in quinine RTs for either lean or obese individuals. This limited evidence suggests that bitter taste sensitivity might not be regulated by diurnal or circadian rhythms, perhaps because bitter taste sensitivity is not a strong influencer on meal patterns,^50–51^ but rather a warning sign for potentially hazardous substances. However, the research is limited to a Japanese population, and further evidence is needed to determine whether patterns in bitter taste sensitivity emerge in other populations.

### Daily patterns in umami taste sensitivity

Two studies have assessed daily patterns in umami sensitivity, both from the same research group in Japan (methods outlined in Daily patterns in sweet taste sensitivity section).^30–31^ Neither study reported overall patterns for monosodium glutamate RTs, but 1 study did observe a difference in RTs between eating protocols.^30^ Participants who were only provided with 1 meal (dinner) had lower RTs at 1700 h and 1900 h compared with participants provided with 2 or 3 meals.

A study in mice measured expression of umami taste receptor genes *TAS1R1* and *TAS1R3* in lingual tissue at 0700 h and 1900 h.^48^*TAS1R1* expression was higher at 1900 h than at 0700 h, but there was no significant difference in *TAS1R3* expression between the time points. This data suggest greater umami function in the evenings, which could represent the readying for food intake during the dark hours, similar to that which was reported by a separate study on sweet taste receptor expression.^49^ Since sweet and umami taste receptors share subunits, it is logical that there are similarities in their regulatory patterns.

Overall, there appears to be some evidence for umami taste sensitivity being regulated by food intake in humans^30^ and mice.^48^ However, further research with controlled eating protocols is necessary in order to determine the true diurnal or circadian rhythms occurring in umami taste sensitivity.

### Daily patterns in fat taste sensitivity

No studies have assessed daily patterns of fat taste sensitivity in humans, but 2 studies in mice have measured daily patterns in the expression of fat taste receptor genes. One study assessed *CD36* and *FFAR4* expressions in papillae tissue in lean and obese mice (methods outlined in the Daily patterns in sweet taste sensitivity section).^49^ Patterns of *CD36* expression differed between lean and obese mice, with lean mice showing 2 peaks in expression at 0600 h and 1800 h, and subdued expression at other times. Conversely, the obese mice demonstrated a single peak in *CD36* expression at 0200 h. The overall patterns of *FFAR4* expression were similar between lean and obese mice, although displaced to the opposite times of the day/night. For example, the lean mice *FFAR4* expression peaked at 1800 h and fell at 0200 h, while the obese mice *FFAR4* expression peaked at 0200 h and fell at 1800 h. These gene expressions in the obese mice seemed to be counterintuitive to what might be expected with typical eating behavior, and perhaps demonstrates dysregulation of taste as a marker of irregular food intake in the obese state.

In another study, *CD36* and *FFAR4* expressions were measured in mice that were given free access to standard chow.^24^ The gene expressions were measured every 3 hours across 24 hours. *CD36* expression decreased during the dark hours and increased during the light hours, with a peak at 1600 h, while *FFAR4* expression showed a contrary pattern of increasing during the dark hours and decreasing during the light hours, with a peak at 0400 h. Furthermore, this study reported that fasting upregulated CD36 protein levels, or in other words, increases in food intake decreases the number of active CD36 receptors. This was not observed for FFAR4 protein levels, which were not affected by short-term food intake. This matches previous findings in humans, in whom CD36 is involved in the regulation of short-term food intake in response to acute oral fat exposure, and FFAR4 is involved in the regulation of long-term dietary management,^19^ and these receptors may perform different but complementary roles in fat taste function and signaling.^52^

Research on fat taste is in a relatively early stage compared with other taste modalities. Early evidence in mice suggests that fat taste is regulated by daily patterns, perhaps in response to food intake, although it is still not entirely clear whether this would translate to humans. Importantly, the patterns differ between lean and obese mice,^49^ which might provide insight into the pathophysiology of metabolic disease. Further research is needed in this area to understand more about the drivers of fat intake.

## MONTHLY (MENSTRUAL) PATTERNS

The menstrual cycle is the oscillation of female sex hormones in females of reproductive age. This cycle has defined temporal phases that repeat over 28 days, on average, but can range anywhere from 21 days to 35 days.^53^ It is divided into 2 main phases: the follicular phase (FP), occurring pre-ovulation, and the luteal phase (LP), occurring post-ovulation. However, these broad categories are not nuanced enough to identify the hormonal fluctuations occurring throughout the cycle. Definitions of the number of days of the menstrual cycle on which each phase occurs is not consistent between studies, and of course this would differ between individuals as well. The timing may even differ between cycles within an individual, making phase estimates difficult and inconsistent to research. A range of methods have been developed to determine menstrual phase, ranging in quality and accuracy.^54^ Most studies included in this review have used the forward-count method, which does not account for individual differences and is largely inaccurate.^54^ For the purposes of review, strict definitions of menstrual phases are needed to ensure consistent comparisons between studies, where biochemical markers are absent. The following definitions are used to estimate menstrual phases based on number of days post-menstruation in a 28-day cycle, with day 1 being the onset of menses (some studies count this as day 0)^54^:

- *Follicular phase* (FP): day 1 until day 14.
  - *Menses or menstruation phase* (MP): day 1 until day 5. Characterized by bleeding and low estrogen and progesterone levels.
  - *Proliferative phase* (PP): day 6 until day 13. Characterized by gradually increasing estrogen while progesterone remains low.
  - *Ovulatory phase* (OP): day 14 until day 15. Characterized by low estrogen and progesterone levels, and a rapid spike in luteinizing hormone level.
- *Luteal phase (LP): day 16 until day 28. Characterized by gradually increasing estrogen and progesterone levels during the early LP, peaking during the mid-LP, and decreasing during the late LP. Since the different phases of the LP cannot be determined based on forward-counting, all days measured from 16 to 28 are categorized together as LP.*

It should be acknowledged that these cycles may not be present for all females. Females with anovulatory phases or females using hormonal contraceptive medications will not exhibit the hormonal patterns of a regular menstrual cycle.

Monthly patterns of food intake in females due to changes in sex hormones during the menstrual cycle are well researched.^55^ In general, energy intake appears to be greatest during the LP and at its lowest immediately preceding OP. Drivers of this phenomenon is likely mediated by estrogen and progesterone, offering some degree of regulatory feedback on a range of physiological mechanisms associated with eating behavior and appetite, where estrogen tends to inhibit appetite while progesterone stimulates appetite.^56–57^

There is some evidence for sex hormone–mediated changes in taste function, which may be 1 potential driver of appetite and food choice that modulates eating behavior during the menstrual cycle. One study has shown that patterns of taste sensitivity for multiple tastes was more closely associated with progesterone levels than with the number of days or estimated phases of the menstrual cycle.^32^ Few studies have measured changes in taste function in men or amenorrheic (eg, postmenopausal or anovulatory) females. This is an important academic point, as it would differentiate whether the monthly patterns of taste functioning and food intake are solely mediated by female sex hormones or whether there are other biological clocks that are linked to these patterns.

In this section, tastes are presented separately. Only the basic tastes have been studied, so alimentary tastes will not be reviewed.

### Monthly patterns in sweet sensitivity

Seven studies have assessed monthly patterns in sweet taste sensitivity. A summary of these studies is presented in Table 1.^32–34^^,^^58–61^ Four studies reported patterns in sweet taste sensitivity being associated with the menstrual cycle in females,^32–34^^,^^58^ while 3 studies did not observe patterns.^59–61^ Three studies used forward-counting as the phasing method,^58–60^ 3 studies used biochemical assessment,^32^^,^^34^^,^^61^ and 1 study did not state the phasing method.^33^ However, the outcomes of the studies did not seem consistent with the phasing methods.

**Table 1:** Summary of studies assessing monthly patterns in sweet taste sensitivity

Author	Tastant	n	Age	Inclusion criteria for females	Phasing method	Days of testing^a^	Summary of results
**Detection threshold**							
Than et al (1994)^58^	Sucrose	14 F 13 M	Range: 18 y–24 y	Not taking OC, regular menstrual cycle (26 d–30 d) for the previous 6 cycles	Forward counting	Days 0–5 (MP) Days 8–12 (PP) Days 17–23 (LP)	PP thresholds were lower than MP and LP thresholds in F; thresholds did not change in M
Alberti-Fidanza et al (1996)^32^	Sucrose	8 F	Range: 23 y–37 y	Not taking OC for 3 months, regular menstrual cycle, no severe premenstrual syndrome	Radioimmunoassay of serum hormones	Day 1 (not reported) Day 7 (PP) Day 14 (OP) Day 21 (LP)	OP thresholds were lower than PP and LP thresholds; thresholds were negatively correlated with estradiol levels
**Recognition threshold**							
Alberti-Fidanza et al (1996)^32^	Sucrose	8 F	Range: 23 y–37 y	Not taking OC for 3 months, regular menstrual cycle, no severe premenstrual syndrome	Radioimmunoassay of serum hormones	Day 1 (not reported) Day 7 (PP) Day 14 (OP) Day 21 (LP)	Slight decrease in thresholds from PP to OP and LP, although the difference was not significant
Kuga et al (1999)^59^	Sucrose	30 F	Mean years ± unknown unit of variation: 29.1 ± 5.6	Not taking OC, regular menstrual cycle (25 d–34 d)	Forward counting	Days 5–10 (FP) Days 20–25 (LP)	No difference in thresholds between FP and LP
Rahul et al (2014)^33^	Glucose	50 F	Range: 18 y–20 y	Not taking OC, regular menstrual cycles (20 d–30 d) for the previous 3 cycles	Not stated	Days 1–6 (MP) Days 7–14 (PP) Days 15–28 (LP)	PP thresholds were lower than LP. No differences between MP and PP, or between MP and LP
Nagai et al (2015)^60^	Sucrose	40 F 30 M	Mean years ± SE: 20.7 ± .3	Not taking OC for 2 months	Forward counting	Not controlled 22 F were in LP and 28 F were in FP	No difference in thresholds between M, F in LP, and F in FP
**Suprathreshold intensity**							
Barbosa et al (2015)^61^	Sucrose	70 F	Mean years ± SD: 23.46 ± 5.29	Regular menstrual cycle (22 d–35 d)	Forward counting; enzyme-linked immunosorbent assay of serum/plasma hormones during LP used as confirmation	Days 10–12 (PP) Days 24–27 (LP)	No difference in sensitivity between PP and LP
Stanić et al (2021)^34^	Sucrose	14 pmW 10 ocW 8 aoW 21 1mcW 29 2mcW 17 M	Median years (IQR) pmW: 56.55 (3.45) ocW: 24.51 (1.50) aoW: 20.56 (16.26) 1mcW: 27.27 (7.87) 2mcW: 27.56 (12.49) M: 24.02 (6.24)	1mcW and 2mcW groups had regular menstrual cycle (22 d–35 d) for the previous 6 cycles	Self-administered urinary LH test to determine OP	In 1mcW and 2mcW groups: FP, OP, LP, and late LP In other groups: days equivalent to FP, OP, and LP	In the ocW group, sensitivity was higher during LP equivalent compared with FP equivalent. No other changes to sensitivity observed within other groups Across groups during the FP (or equivalent), sensitivity was higher in M than in pmW and ocW, and sensitivity was higher in 2mcW than in ocW.

*Abbreviations:* F, females; FP, follicular phase; LP, Luteal phase; M, males; MP, menstrual phase; OC, oral contraceptives; OP, ovulation; PP, proliferative phase; pmW, postmenopausal women; ocW, women taking oral contraceptives; aoW, women with anovulatory cycle; 1mcW, women across 1 menstrual cycle; 2mcW, women across 2 menstrual cycles.

Two studies assessed sweet taste DTs, with consistent results^32^^,^^58^: DTs were observed to be higher during the MP and the LP, and lower during the PP and the OP. One of these studies also noted that DTs were negatively associated with serum estradiol levels,^32^ marking this hormone as a potential regulator of sweet taste sensitivity. Men did not show any changes in DTs across the same period,^58^ which makes sense, as men do not exhibit patterns in serum estradiol levels.

Studies assessing sweet taste RTs have shown mixed results. One study reported higher RTs in the PP and lower RTs in the LP,^32^ while another study reported the opposite, with RTs being lower in the PP and higher in the LP.^33^ Two more studies found no differences in RTs between phases^59–60^ or between sexes.^60^ These differences may be attributed to lack of accuracy when reporting the menstrual cycle phase, as most studies used self-reporting by the participants to track the cycles. In addition, each study had slightly different criteria for calculating the menstrual phases.

Two studies have assessed monthly patterns in sweet taste ST intensity: 1 reported no patterns^61^ while the other reported a general pattern of raised ST intensity during the mid-LP and decreased ST intensity during the FP, although the difference was not statistically significant.^34^ The latter study also assessed amenorrheic subgroups, defined as post-menopausal women (pmW), women using oral contraceptives (ocW), and women with an anovulatory cycle (aoW).^34^ They observed a pattern in ocW in the same direction as what was observed in eumenorrheic women, with ST intensity being higher during days equivalent to the LP and lower during days equivalent to the FP. In addition, ocW had lower overall ST intensity than eumenorrheic women in all phases. This is an interesting finding, as oral contraceptive medication should eliminate, or at least greatly reduce, hormonal changes throughout the menstrual cycle. The ocW were on monophasic oral contraceptives, which contain estrogen and progesterone. These hormones are likely to have altered taste perception, although it is unclear why a pattern of taste sensitivity would have emerged.

Overall, there appears to be mixed results for menstrual patterns in sweet taste sensitivity across the 3 taste measures. Patterns in DTs were congruent with expectations of energy intake across the menstrual cycle, with higher DTs during LP,^32^^,^^58^ which is typically when greater energy intake is observed.^55^ Thus, the DT may be a better marker of hormone-mediated changes in sweet taste sensitivity, compared with the RT or ST intensity.

### Monthly patterns in salty taste sensitivity

Seven studies have assessed the monthly patterns in salty taste sensitivity. A summary of these studies is presented in Table 2.^32–34^^,^^59^^,^^61–63^ Four studies reported patterns in salty taste sensitivity associated with the menstrual cycle in females (Alberti-Fidanza et al 1996; Byun et al 2001; Rahul et al 2014; Stanić et al 2021),^32–34^^,^^62^ while 3 studies did not observe such patterns.^59^^,^^61^^,^^63^ Three studies used forward-counting as the phasing method,^59^^,^^62–63^ 3 studies used biochemical assessment,^32^^,^^34^^,^^61^ and 1 study did not state the phasing method.^33^ As with sweet taste sensitivity, phasing methods did not appear to be an influencer of the study outcomes.

**Table 2:** Summary of the studies assessing monthly patterns in salty taste sensitivity

Author	n	Age	Inclusion criteria for females	Phasing method	Day(s) of Testing^a^	Summary of results
**Detection threshold**						
Alberti-Fidanza et al (1996)^32^	8 F	Range: 23 y–37 y	Not taking OC for 3 months, regular menstrual cycle, no severe premenstrual syndrome	Radioimmunoassay of serum hormones	Day 1 (not reported) Day 7 (PP) Day 14 (OP) Day 21 (LP)	Differences between phases not stated Thresholds were negatively correlated with progesterone levels.
Byun et al (2001)^62^	11 F	Mean: 23.2 y Range: 20 y–27 y	Regular menstrual cycle (28 d–30 d)	Forward counting	Days 1–7 (MP) Days 8–14 (PP) Days 15–21 (LP)	MP thresholds tended to be lower than PP and LP thresholds, but this difference was not significant.
**Recognition threshold**						
Alberti-Fidanza et al (1996)^32^	8 F	Range: 23 y–37 y	Not taking OC for 3 months, regular menstrual cycle, no severe premenstrual syndrome	Radioimmunoassay of serum hormones	Day 1 (not reported) Day 7 (PP) Day 14 (OP) Day 21 (LP)	Differences between phases not stated. Thresholds were negatively correlated with progesterone levels.
Kuga et al (1999)^59^	30 F	Mean years ± unknown unit of variation: 29.1 ± 5.6	Not taking OC, regular menstrual cycle (25 d–34 d)	Forward counting	Days 5–10 (FP) Days 20–25 (LP)	No difference in thresholds between FP and LP
Rahul et al (2014)^33^	50 F	Range: 18 y–20 y	Not taking OC, regular menstrual cycles (20 d–30 d) for the previous 3 cycles	Not stated	Days 1–6 (MP) Days 7–14 (PP) Days 15–28 (LP)	MP thresholds were lower than FP and LP.
**Suprathreshold intensity**						
Frye and Demolar (1993)^63^	49 F 31 M	Range: 17 y–22 y	Not taking OC, regular menstrual (22 d–31 d) cycle for the previous 6 cycles	Forward counting	Not controlled Number of F in each phase not stated	No difference in sensitivity between phases or sex
Barbosa et al (2015)^61^	70 F	Mean ± SD years: 23.46 ± 5.29	Regular menstrual cycle (22 d–35 d)	Forward counting; enzyme-linked immunosorbent assay of serum/plasma hormones during LP used as confirmation	Days 10–12 (PP) Days 24–27 (LP)	No difference in sensitivity between PP and LP
Stanić et al (2021)^34^	14 pmW 10 ocW 8 aoW 21 1mcW 29 2mcW 17 M	Median years (IQR) pmW: 56.55 (3.45) ocW: 24.51 (1.50) aoW: 20.56 (16.26) 1mcW: 27.27 (7.87) 2mcW: 27.56 (12.49) M: 24.02 (6.24)	1mcW and 2mcW groups had regular menstrual cycle (22 d–35 d) for the previous 6 cycles	Self-administered urinary LH test to determine ovulation	In 1mcW and 2mcW groups: FP, OP, LP, and late LP In other groups: days equivalent to FP, OP, and LP	In the 1mcW group, sensitivity was higher during LP compared with FP. No other changes to sensitivity observed within other groups. Across groups, sensitivity was higher in M than in pmW during FP equivalent and OP equivalent measures.

All studies used sodium chloride as a tastant. ^a^Number of days from the first day of the last menstrual period. *Abbreviations:* F, females; FP, follicular phase; LP, Luteal phase; M, males; MP, menstrual phase; OC, oral contraceptives; OP, ovulation; PP, proliferative phase; pmW, postmenopausal women; ocW, women taking oral contraceptives; aoW, women with anovulatory cycle; 1mcW, women across 1 menstrual cycle; 2mcW, women across 2 menstrual cycles.

One study reported that DTs tended to be lower during MP than during PP and LP, although the difference between the phases was not statistically significant.^62^ This was observed in a sample of 11 females, which may have been underpowered to show true effects of the menstrual cycle on salty taste sensitivity. Another study reported that DTs were negatively associated with progesterone levels in 8 females.^32^ While this study did not state any temporal patterns in salty taste DT, it can be assumed that DTs follow a similar pattern to the temporal regulation of progesterone across the menstrual cycle.

Similar to DTs, salty taste RTs were reported to be negatively associated with progesterone levels, demonstrating that monthly patterns in RT may have been present in a small sample of female participants.^32^ Another study reported significantly lower RTs during MP, compared with FP and LP, which matches the DT patterns seen in an earlier study.^62^ One study, however, reported no observable monthly pattern in RTs in 30 females.^59^

Two studies assessing ST intensity reported no monthly patterns in salty taste sensitivity in males or females,^61^^,^^63^ while another study reported a monthly pattern in eumenorrheic females.^34^ Eumenorrheic females showed higher ST intensity during the LP compared with the FP, similar to what was observed for sweet taste. Additionally, ocW showed a trend for increasing ST intensity in the days equivalent to LP, although this was not statistically significant.

While 4 out of the 7 studies assessing monthly patterns in salty taste sensitivity reported an observed pattern, the patterns described were inconsistent with one another and incongruent with the expectations of energy intake.^55^ Salty taste sensitivity is likely mediated by female sex hormones to some degree, but it is difficult to determine any associated dietary patterns, given the available data. One study has reported changes in plasma sodium balance across the menstrual cycle, but this was not accompanied by changes in dietary sodium intake,^64^ so salty taste sensitivity may not be a target for sex hormone mediation.

### Monthly patterns in sour taste sensitivity

Four studies have assessed the monthly patterns in sour taste sensitivity. Two studies reported patterns in sour taste sensitivity associated with the menstrual cycle in females,^34^^,^^61^ while 2 studies did not observe patterns.^33^^,^^59^ Of these latter studies that did not observe patterns, 1 measured tartaric acid RTs^59^ and 1 measured citric acid RTs.^33^ No studies to date have assessed monthly patterns in sour taste DT. One study used forward-counting as the phasing method,^59^ 2 studies used biochemical assessment,^34^^,^^61^ and 1 study did not state the phasing method.^33^ The studies that used biochemical assessment to phase participants were also the only studies that reported menstrual patterns in sour taste sensitivity.

Two studies assessing ST intensity reported conflicting monthly patterns in sour taste sensitivity in females.^34^^,^^61^ One study reported higher ST intensity during the FP,^61^ while the other study showed higher ST intensity during the LP compared with the FP.^34^ Differences in patterns may be due to differences in study methodologies. For example, the former study excluded participants if they could not correctly complete a taste-ranking task, which may have caused a bias towards those who have greater taste perception. Also, 1 study used citric acid^61^ and the other used ascorbic acid^34^ as tastants. Additionally, the latter study showed a trend for increasing ST intensity in ocW during the days equivalent to LP, similar to sweet and salty taste trends, although this was not statistically significant.^34^

Few studies have assessed menstrual patterns in sour taste sensitivity, and no studies have assessed DTs. In addition, it appears that differences in methodologies may have influenced the outcomes, as only the more accurate biochemical phasing methods were able to detect menstrual patterns in sour taste sensitivity. Therefore, it is difficult to make definitive conclusions on whether monthly patterns in sour taste sensitivity exist, and more research is needed.

### Monthly patterns in bitter taste sensitivity

Seven studies have assessed the monthly patterns in bitter taste sensitivity. A summary of these studies is presented in Table 3.^32–34^^,^^59^^,^^61^^,^^65–66^ Two studies reported patterns in bitter taste sensitivity associated with the menstrual cycle in females,^33^^,^^66^ while 5 studies did not observe patterns.^32^^,^^34^^,^^59^^,^^61^^,^^65^ Three studies used forward- and/or backward-counting as the phasing method,^59^^,^^65–66^ 3 studies used biochemical assessment,^32^^,^^34^^,^^61^ and 1 study did not state the phasing method.^33^ The outcomes for each study did not appear to be affected by the phasing method.

**Table 3:** Summary of the studies assessing monthly patterns in bitter taste sensitivity

Author	Tastant	n	Age	Inclusion criteria for females	Phasing method	Day(s) of testing^a^	Summary of results
**Detection threshold**							
Beiguelman (1964)^65^	Phenylthiourea	100 F	Not stated	Not stated	Combination of forward and backward counting	MP 15 days before or after MP (non-MP)	No difference in thresholds between MP and non-MP
Glanville and Kaplan (1965)^66^	6-n-propylthiouracil and quinine sulphate	19 F	Mean: 20.7 y Range: 19 y–27 y	Not taking drugs for the duration of the study	Backward counting	Day –9 to –5 (LP) Day –1 to +4 (MP) Day +6 to +10 (PP)	MP thresholds were lower than LP and PP
Alberti-Fidanza et al (1996)^32^	Quinine sulphate	8 F	Range: 23 y–37 y	Not taking OC for 3 months, regular menstrual cycle, no severe premenstrual syndrome	Radioimmunoassay of serum hormones	Day 1 (not reported) Day 7 (PP) Day 14 (OP) Day 21 (LP)	No difference in thresholds between phases Thresholds negatively correlated with progesterone levels
**Recognition threshold**							
Alberti-Fidanza et al (1996)^32^	Quinine sulphate	8 F	Range: 23 y–37 y	Not taking OC for 3 months, regular menstrual cycle, no severe premenstrual syndrome	Radioimmunoassay of serum hormones	Day 1 (not reported) Day 7 (PP) Day 14 (OP) Day 21 (LP)	No difference in thresholds between phases Thresholds negatively correlated with progesterone levels in smokers only
Kuga et al (1999)^59^	Quinine hydrochloride	30 F	Mean years ± unknown unit of variation: 29.1 ± 5.6	Not taking OC, regular menstrual cycle (25 d–34 d)	Forward counting	Days 5–10 (FP) Days 20–25 (LP)	No difference in thresholds between FP and LP
Rahul et al (2014)^33^	Quinine sulphate	50 F	Range: 18 y–20 y	Not taking OC, regular menstrual cycles (20 d–30 d) for the previous 3 cycles	Not stated	Days 1–6 (MP) Days 7–14 (PP) Days 15–28 (LP)	LP thresholds were lower than MP and FP.
**Suprathreshold intensity**							
Barbosa et al (2015)^61^	Caffeine	70 F	Mean ± SD years: 23.46 ± 5.29	Regular menstrual cycle (22 d–35 d)	Forward counting; enzyme-linked immunosorbent assay of serum/plasma hormones during LP used as confirmation	Days 10–12 (PP) Days 24–27 (LP)	No difference in sensitivity between PP and LP
Stanić et al (2021)^34^	Quinine hydrochloride	14 pmW 10 ocW 8 aoW 21 1mcW 29 2mcW 17 M	Median (IQR) pmW: 56.55 (3.45) ocW: 24.51 (1.50) aoW: 20.56 (16.26) 1mcW: 27.27 (7.87) 2mcW: 27.56 (12.49) M: 24.02 (6.24)	1mcW and 2mcW groups had regular menstrual cycle (22 d–35 d) for the previous 6 cycles	Self-administered urinary LH test to determine ovulation	In 1mcW and 2mcW groups: FP, OP, LP, and late LP In other groups: days equivalent to FP, OP, and LP	No differences in sensitivity within or between any groups and phases

a Number of days from the first day of the last menstrual period. *Abbreviations:* F, females; FP, follicular phase; LP, Luteal phase; M, males; MP, menstrual phase; OC, oral contraceptives; OP, ovulation; PP, proliferative phase; pmW, postmenopausal women; ocW, women taking oral contraceptives; aoW, women with anovulatory cycle; 1mcW, women across 1 menstrual cycle; 2mcW, women across 2 menstrual cycles.

Two of the earliest studies to assess monthly patterns in taste sensitivity reported conflicting results. A 1964 study assessing 100 female participants reported no differences in the DTs between MP and “non-MP”, although the article is unclear about the nature of the phases measured during the non-MP time frame.^65^ A 1965 study did observe a pattern in a sample of 19 female participants, with lower DTs being observed during MP, compared with LP and PP.^66^ One reason for this difference may be the use of different tastants, with the former study using phenylthiourea^65^ and the latter study using both 6-n-propylthiouracil and quinine sulphate.^66^ There are at least 25 bitter receptors in humans,^9^ and each tastant may act through different bitter perception pathways, which may explain the differences between tastants. A more recent study assessed monthly patterns in DTs in 8 female participants, using quinine sulphate, and although they did not observe any patterns, they did show a negative association between DT and progesterone.^32^ As with other tastes, this demonstrates the potential for female sex hormones to regulate taste systemically.

Three studies have assessed monthly patterns in bitter taste RTs, using salts of quinine as the tastant, with mixed findings. One study reported that RTs were lower during LP compared with MP and FP,^33^ 1 study reported no patterns,^59^ and the other study reported no patterns overall but observed a negative association between RT and progesterone in a subset of participants who were smokers.^32^ This finding is dubious, as smokers are usually excluded from taste studies because smoking may diminish taste sensitivity. Nonetheless, the result is still interesting, and in line with progesterone-mediated taste regulation found with other taste modalities and dimensions.

Two studies have assessed monthly patterns in bitter taste ST intensity.^34^^,^^61^ In both studies, no monthly patterns in ST intensity were reported in either males or females.

Five of the 7 studies assessing monthly patterns in bitter taste sensitivity did not observe patterns. The 2 studies that did observe patterns either used outdated methodology^66^ or did not disclose the phasing methods, so the true phase could not be determined.^33^ It is suggested that the menstrual cycle probably does not regulate bitter taste sensitivity. This is a logical conclusion, as bitter taste is unlikely to be regulated temporally, since it is not strongly associated with energy intake^50–51^ and would serve little purpose in the dietary changes associated with preparation for pregnancy.

## YEARLY (SEASONAL) PATTERNS

Traditionally, food intake patterns would have been driven by seasonal food availability across a year. Changes in climates, timing of harvests, and ability to store foods long term would have all had an impact on human yearly food intake patterns.^67^ Today, advances in agriculture and a globalized food supply have led to much greater food availability throughout the entire year, even of foods that would normally be “out-of-season.” Despite this, yearly patterns in food intake are still evident.^68^ Physiological adaptations to seasonal food intake over thousands of years has had an impact on the evolution of regulatory mechanisms that operate on a seasonal calendar, including physiological, neurochemical, and hormonal mechanisms.^69^ These adaptations are in response to the quality of foods available (types of foods available in cooler vs warmer seasons) as well as the quantity of foods, where periods of feast and famine may line up with seasonal shifts. Drivers of food intake to mitigate energy loss during fasting or famine would have been necessary for the survival of human communities.

In general, food and energy intake are greater in winter compared with summer across multiple populations.^70–72^ While there are many factors involved, 1 driver is related to changes in flavor perception and hedonic preferences. For example, there tends to be a greater preference for savory foods during cooler periods.^73^ It should also be acknowledged that changes in food preferences could be due to conditioning and social norms, such as seasonal holidays.^74–75^ Given that, at least for Western cultures, celebratory holidays occur during the same calendar months around the world, even though seasons may differ (ie, in the Northern hemisphere vs the Southern hemisphere), the seasonal patterns of different nations should be considered during interpretation of the data.

Few studies have assessed seasonal patterns in gustation, despite seasonal patterns of food intake being well documented.^68^ Not enough data exists for each taste modality, so this section of the review is not categorized by taste. Also, only the basic tastes have been studied, so alimentary tastes will not be discussed.

A 1968 study measured salty taste detection (called “uncertain”) and recognition (called “certain”) thresholds in 3 young male students from the United States over 120 consecutive days (days 1–5 being excluded from the analysis).^35^ Thresholds were measured at the same time of day, each day. The study was conducted during the “fall months,” although it is unclear whether the study dates spilled into summer and/or winter. Regardless, this study provides some insight into the changes in sensitivity across approximately 4 months and across at least 1 change in season. Three patterns emerged in salty taste DT across 115 days: One participant showed a gradual increase in DTs; 1 participant showed a large decrease in DTs; and 1 participant showed a relatively stable DTs. The RT data was not reported, but the authors state that the day-to-day variation in RTs were similar to that in the DTs, “but at a higher molarity”. Although inconsistent, this is an important early finding in the long-term patterns in taste sensitivity, as it demonstrates that patterns differ between individuals. Differences in these patterns may be markers of eating behavior or of health status, although investigating this was outside the scope of the study being reviewed.

**Figure 2:**
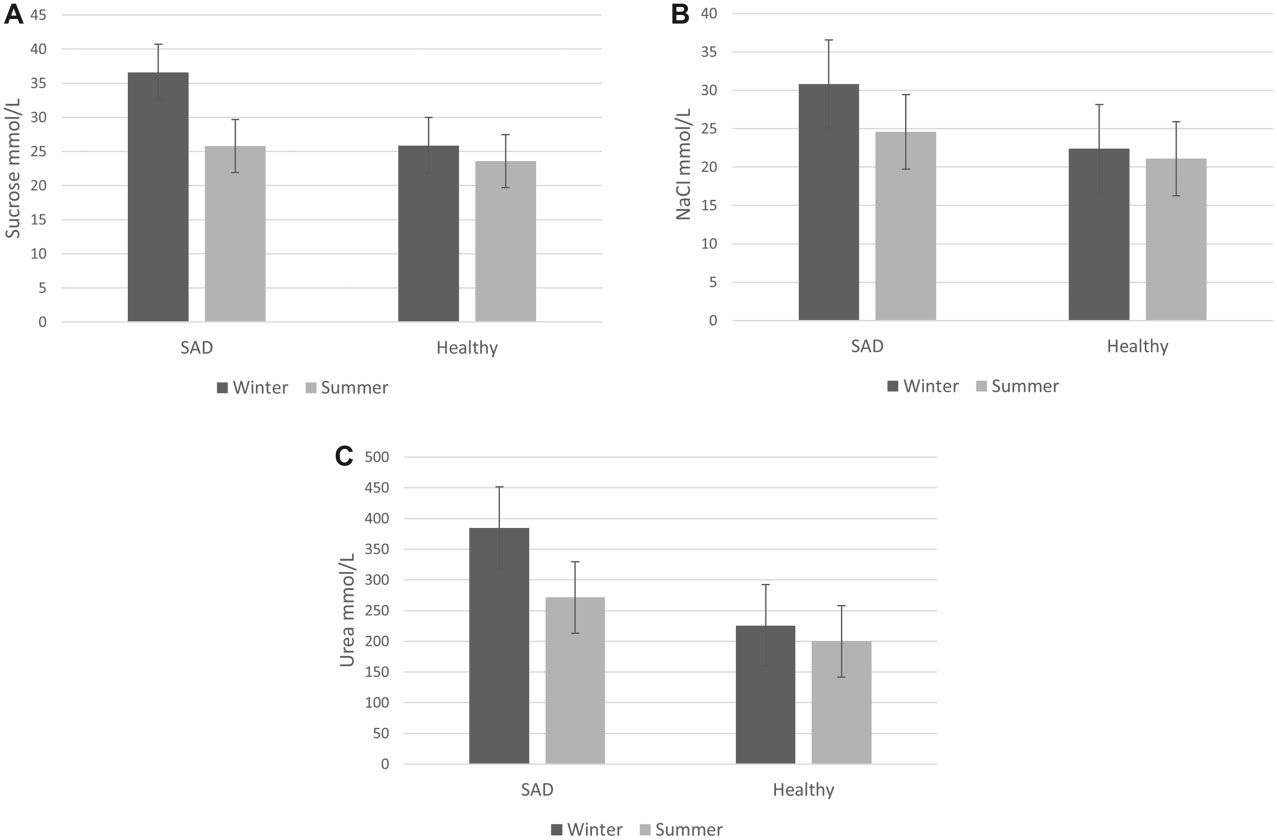
**Sweet taste (A), salty taste (B), and bitter taste (C) detection thresholds in winter and summer in healthy participants and participants with seasonal affective disorder (SAD).** Data from Arbisi et al. (1996).^36^

A 1996 study conducted in the United States compared DTs and RTs for sweet taste (sucrose), sour taste (hydrochloric acid), salty taste (sodium chloride), and bitter taste (urea) between winter and summer in 23 healthy participants and 25 participants diagnosed with seasonal affective disorder (SAD).^36^ For sweet and salty tastes, DTs were lower in summer compared with winter (Figure 2).^36^ Furthermore, in winter only, participants with SAD had higher sweet taste, salty taste, and bitter taste DTs compared with healthy participants (Figure 2). This suggests that seasonal-mediated changes in taste sensitivity may only affect certain individuals or susceptible subgroups. Similarly, it has been reported that people with SAD have lower odor DTs,^76^ so SAD may subdue chemosensation holistically, rather than being specific to taste. No patterns were observed in the sour taste DTs, or in the RTs for any of the basic tastes.

The final study to assess seasonal patterns in taste sensitivity is only available as an abstract presented at a conference.^77^ An equivalent journal article presenting the full study does not appear to be available. Due to the limited research available on this topic, this review has presented the findings of this study abstract out of interest, but these findings have not been peer-reviewed and interpretations should be cautious. In this study, the RTs for sweet taste (sucrose), salty taste (sodium chloride), sour taste (tartaric acid), and bitter taste (quinine) were measured in 22 healthy young females during winter, summer, and the following winter.^77^ All RT measures were assessed using filter paper disks soaked in the respective solutions. Salty taste RTs were measured a second time using filter paper strips embedded with sodium chloride. No differences between seasons were observed for any of the RTs measured with the taste disks. For the salty taste RTs measured with the taste strips, RTs decreased from winter 1 to summer in 17 out of 22 participants and increased from summer to winter 2 in 16 of 22 participants (mean RT ± unknown unit of variation: winter 1 (.16 ± .02 mg/cm^2^); summer (.08 ± .01 mg/cm^2^); winter 2 (.15 ± .02 mg/cm^2^). Interestingly, 5 to 6 participants appeared to be resistant to seasonal-mediated changes in salty taste RTs. Also, differences in salty taste RTs were noted for 1 methodology but not for another, so it is important to ensure valid and rigorous methods are used to ensure accurate results.

Of the few studies that have been conducted, some seasonal patterns have emerged in sweet,^36^ bitter,^36^ and salty tastes.^35–36^^,^^77^ These patterns appear to present differently across individuals, with some evidence that there may be individuals who are more susceptible to seasonal-mediated changes. This should be investigated further, as 1 study has demonstrated that the susceptible group were people with SAD, who may already be at a greater health risk than healthy individuals.^36^ As noted for daily patterns in taste sensitivity, differences in patterns between individuals may provide some insights into current health states, or predisposition to certain health states or diseases. This warrants further investigation in larger samples.

## CONCLUSION

This review presents an overview of the literature on patterns in taste sensitivity across different temporal dimensions. Daily, monthly, and yearly patterns in taste sensitivity have been observed, but the relationships between taste sensitivity and corresponding food intake patterns vary. Other cyclical patterns in taste sensitivity may also exist (eg, weekly food intake patterns^78^), but the evidence for matching gustatory patterns is lacking.

A notable bias in the literature is that studies assessing more than 1 taste modality usually observed patterns for multiple tastes, which may be due to the methods used or participant sampling. Inconsistencies in methodologies, particularly the use of different tastants for bitter and sour tastes, further complicate the interpretation of the results. Despite these limitations, taste sensitivity patterns have promising potential as markers of nutrition and food intake.

Daily patterns in taste sensitivity are the patterns most strongly supported by evidence, particularly for sweet taste, fat taste, and umami taste. Given the strong relationship between these 3 taste modalities and dietary intake, this may add to the body of evidence for the role of the gustatory system in the regulation of dietary intake. However, some evidence for these patterns comes from rodent studies. Mice are nocturnal mammals, and thus have different diurnal patterns to humans, so their applicability to human populations remains uncertain. Nevertheless, the fact that patterns exist in other mammals suggests that similar patterns could also be exhibited in humans as well, albeit at different time points.

Monthly patterns in taste sensitivity are less apparent for most tastes. The 1 exception was for sweet taste, which seemed to be related to changes in food intake across the menstrual cycle. Differences in phasing methods made it difficult to make fair comparisons between studies, with sour taste sensitivity appearing to be influenced by the methodology used. More consistent standards for menstrual phasing are needed for sensory research.

Yearly patterns have had the least amount of research conducted, but weak evidence suggests that there are potential nonspecific changes in gustatory perception across the seasons. Alimentary tastes have not been investigated in either of these temporal dimensions, but research on umami taste, fat taste, and carbohydrate taste may provide insights into the role of taste in dietary intake across monthly and yearly cycles.

Overall, taste sensitivity may have the potential to inform dietary patterns and improve personalized approaches to dietary management. Measures of taste sensitivity are easy to conduct, and new technologies for capturing biomarkers of gustation make taste function assessment more rapid and accurate.^23^ However, the literature has notable gaps, particularly around alimentary tastes, and further research is needed to expand the understanding of patterns in taste sensitivity and their relationship to dietary health.

## References

[1] Harnischfeger F , Dando R. Obesity-induced taste dysfunction, and its implications for dietary intake. Int J Obes (Lond). 2021;45:1644–1655. doi: 10.1038/s41366-021-00855-w 34031530

[2] Dinehart ME , Hayes JE, Bartoshuk LM, et al Bitter taste markers explain variability in vegetable sweetness, bitterness, and intake. Physiol Behav. 2006;87:304–313. doi: 10.1016/j.physbeh.2005.10.018 16368118

[3] Bell KI , Tepper BJ. Short-term vegetable intake by young children classified by 6-n-propylthoiuracil bitter-taste phenotype. Am J Clin Nutr. 2006;84:245–251. doi: 10.1093/ajcn/84.1.245 16825702

[4] Kershaw JC , Mattes RD. Nutrition and taste and smell dysfunction. World J Otorhinolaryngol Head Neck Surg. 2018;4:3–10. doi: 10.1016/j.wjorl.2018.02.006 30035256 PMC6051307

[5] Poette J , Mekoué J, Neyraud E, et al Fat sensitivity in humans: oleic acid detection threshold is linked to saliva composition and oral volume. Flavour Fragr J. 2014;29:39–49. doi: 10.1002/ffj.3177

[6] Costanzo A , Orellana L, Nowson C, et al Fat taste sensitivity is associated with short-term and habitual fat intake. Nutrients. 2017;9:781. doi: 10.3390/nu9070781 28726767 PMC5537895

[7] Low JY , Lacy KE, McBride RL, et al The associations between oral complex carbohydrate sensitivity, BMI, liking, and consumption of complex carbohydrate based foods. J Food Sci. 2018;83:2227–2236. doi: 10.1111/1750-3841.14276 30020540

[8] Scott K. Taste recognition: food for thought. Neuron. 2005;48:455–464. doi: 10.1016/j.neuron.2005.10.015 16269362

[9] Gravina SA , Yep GL, Khan M. Human biology of taste. Ann Saudi Med. 2013;33:217–222. doi: 10.5144/0256-4947.2013.217 23793421 PMC6078535

[10] Teng B , Wilson CE, Tu T-H, et al Cellular and neural responses to sour stimuli require the proton channel Otop1. Curr Biol. 2019;29:3647–3656.e5. doi: 10.1016/j.cub.2019.08.077 31543453 PMC7299528

[11] Shekdar K , Langer J, Venkatachalan S, et al Cell engineering method using fluorogenic oligonucleotide signaling probes and flow cytometry. Biotechnol Lett. 2021;43:949–958. doi: 10.1007/s10529-021-03101-5 33683511 PMC7937778

[12] Hartley IE , Liem DG, Keast R. Umami as an ‘alimentary’ taste. A new perspective on taste classification. Nutrients. 2019;11:182. doi: 10.3390/nu11010182 30654496 PMC6356469

[13] Keast R , Costanzo A, Hartley I. Macronutrient sensing in the oral cavity and gastrointestinal tract: alimentary tastes. Nutrients. 2021;13:667. doi: 10.3390/nu13020667 33669584 PMC7922037

[14] Yamaguchi S. The umami taste. In Boudreau JC, ed. Food Taste Chemistry. Washington, DC: American Chemical Society; 1979.

[15] Keast RS , Costanzo A. Is fat the sixth taste primary? Evidence and implications. Flavour. 2015;4:1–7. doi: 10.1186/2044-7248-4-5

[16] Low JY , Lacy KE, McBride RL, et al Evidence supporting oral sensitivity to complex carbohydrates independent of sweet taste sensitivity in humans. PLoS One. 2017;12:e0188784. doi: 10.1371/journal.pone.0188784 29281655 PMC5744938

[17] Liu D , Archer N, Duesing K, et al Mechanism of fat taste perception: association with diet and obesity. Prog Lipid Res. 2016;63:41–49. doi: 10.1016/j.plipres.2016.03.002 27155595

[18] Liu D , Costanzo A, Evans MD, et al Expression of the candidate fat taste receptors in human fungiform papillae and the association with fat taste function. Br J Nutr. 2018;120:64–73. doi: 10.1017/S0007114518001265 29936924

[19] Costanzo A , Liu D, Nowson C, et al A low-fat diet up-regulates expression of fatty acid taste receptor gene *FFAR4* in fungiform papillae in humans: a co-twin randomised controlled trial. Br J Nutr. 2019;122:1212–1220. doi: 10.1017/S0007114519002368 31524116

[20] ISO 5492:2008. Sensory Analysis – Vocabulary. Geneva, Switzerland: International Organization for Standardization; 2008. Available at: https://www.iso.org. Accessed June 22, 2023.

[21] Webb J , Bolhuis DP, Cicerale S, et al The relationships between common measurements of taste function. Chemosens Percept. 2015;8:11–18. doi: 10.1007/s12078-015-9183-x 26110045 PMC4475569

[22] Adler E , Hoon MA, Mueller KL, et al A novel family of mammalian taste receptors. Cell. 2000;100:693–702. doi: 10.1016/S0092-8674(00)80705-9 10761934

[23] Archer NS , Liu D, Shaw J, et al A comparison of collection techniques for gene expression analysis of human oral taste tissue. PLoS One. 2016;11:e0152157. doi: 10.1371/journal.pone.0152157 27010324 PMC4807031

[24] Martin C , Passilly-Degrace P, Gaillard D, et al The lipid-sensor candidates CD36 and GPR120 are differentially regulated by dietary lipids in mouse taste buds: impact on spontaneous fat preference. PLoS One. 2011;6:e24014. doi: 10.1371/journal.pone.0024014 21901153 PMC3162022

[25] Takahata Y , Yoshimoto W, Kuwagaki E, et al Alteration of sweet taste receptor expression in circumvallate papillae of mice with decreased sweet taste preference induced by social defeat stress. J Nutr Biochem. 2022;107:109055. doi: 10.1016/j.jnutbio.2022.109055 35643284

[26] Dey B , Kawabata F, Kawabata Y, et al Bitter taste sensitivity and the expression of bitter taste receptors at different growth stages of chicks. J Poult Sci. 2018;55:204–209. doi: 10.2141/jpsa.0170188 32055176 PMC6756504

[27] Shigemura N , Shirosaki S, Sanematsu K, et al Genetic and molecular basis of individual differences in human umami taste perception. PLoS One. 2009;4:e6717. doi: 10.1371/journal.pone.0006717 19696921 PMC2725291

[28] Martin C , Passilly-Degrace P, Chevrot M, et al Lipid-mediated release of GLP-1 by mouse taste buds from circumvallate papillae: putative involvement of GPR120 and impact on taste sensitivity. J Lipid Res. 2012;53:2256–2265. doi: 10.1194/jlr.M025874 22904345 PMC3465995

[29] Kinnamon SC , Finger TE. Recent advances in taste transduction and signaling. F1000Res. 2019;8:F1000 Faculty Rev-2117. doi: 10.12688/f1000research.21099.1 PMC7059786 32185015

[30] Nakamura Y , Sanematsu K, Ohta R, et al Diurnal variation of human sweet taste recognition thresholds is correlated with plasma leptin levels. Diabetes. 2008;57:2661–2665. doi: 10.2337/db07-1103 18633111 PMC2551675

[31] Sanematsu K , Nakamura Y, Nomura M, et al Diurnal variation of sweet taste recognition thresholds is absent in overweight and obese humans. Nutrients. 2018;10:297. doi: 10.3390/nu10030297 29498693 PMC5872715

[32] Alberti-Fidanza A , Fruttini D, Servili M. Gustatory and food habit changes during the menstrual cycle. Int J Vitam Nutr Res. 1998;68:149–153. 9565832

[33] Rahul K , Santosh W, Swati T, et al Taste recognition threshold in different phases of menstrual cycle. Panacea J Med Sci. 2014;4:45–48.

[34] Stanić Ž , Pribisalić A, Bošković M, et al Does each menstrual cycle elicit a distinct effect on olfactory and gustatory perception? Nutrients. 2021;13:2509. doi: 10.3390/nu13082509 34444669 PMC8401541

[35] Mefferd RB Jr , Wieland BA. Taste thresholds for sodium chloride in longitudinal experiments. Percept Mot Skills. 1968;27:295–315. doi: 10.2466/pms.1968.27.1.295 5685708

[36] Arbisi PA , Levine AS, Nerenberg J, et al Seasonal alteration in taste detection and recognition threshold in seasonal affective disorder: the proximate source of carbohydrate craving. Psychiatry Res. 1996;59:171–182. doi: 10.1016/0165-1781(95)02816-1 8930022

[37] Goetzl FR , Ahokas AJ, Payne JG. Occurrence in normal individuals of diurnal variations in sensitivity of the sense of taste for sucrose. J Appl Physiol. 1950;2:619–626. 15436365 10.1152/jappl.1950.2.11.619

[38] Hammer FJ. The relation of odor, taste, and flicker-fusion thresholds to food intake. J Comp Physiol Psychol. 1951;44:403–411. doi: 10.1037/h0062712 14888733

[39] Fujimura A , Kajiyama H, Tateishi T, et al Circadian rhythm in recognition threshold of salt taste in healthy subjects. Am J Physiol. 1990;259:R931–R935. doi: 10.1152/ajpregu.1990.259.5.R931 2240276

[40] Smeets AJ , Westerterp-Plantenga MS. Oral exposure and sensory-specific satiety. Physiol Behav. 2006;89:281–286. doi: 10.1016/j.physbeh.2006.06.011 16875704

[41] Li T , Zhao M, Raza A, et al The effect of taste and taste perception on satiation/satiety: a review. Food Funct. 2020;11:2838–2847. doi: 10.1039/C9FO02519G 32195512

[42] Spence C. Explaining diurnal patterns of food consumption. Food Qual Prefer. 2021;91:104198. doi: 10.1016/j.foodqual.2021.104198

[43] de Castro JM. Circadian rhythms of the spontaneous meal pattern, macronutrient intake, and mood of humans. Physiol Behav. 1987;40:437–446. doi: 10.1016/0031-9384(87)90028-X 3628541

[44] Booth DA. How nutritional effects of foods can influence people’s dietary choices. In: Barker LM, ed. The Psychobiology of Human Food Selection. Westport, CT: AVI Publishing Company; 1982;67–84.

[45] Halberg F. Chronobiology and nutrition. Contemp Nutr. 1983;8:2. doi: 10.1016/j.neuroscience.2013.08.049

[46] Breslin PA , Izumi A, Tharp A, et al Evidence that human oral glucose detection involves a sweet taste pathway and a glucose transporter pathway. PLoS One. 2021;16:e0256989. doi: 10.1371/journal.pone.0256989 34614010 PMC8494309

[47] Camargo ACB , Castilhos MBM, Conti AC. Perception and sensory acceptance of sweet taste by individuals that who work/study on different shifts. Rev Nutr. 2022;35:e220037. doi: 10.1590/1678-9865202235e220037

[48] O’Brien P , Hewett R, Corpe C. Sugar sensor genes in the murine gastrointestinal tract display a cephalocaudal axis of expression and a diurnal rhythm. Physiol Genomics. 2018;50:448–458. doi: 10.1152/physiolgenomics.00139.2017 29625018

[49] Bernard A , Dastugue A, Maquart G, et al Diet-induced obesity alters the circadian expression of clock genes in mouse gustatory papillae. Front Physiol. 2020;11:726. doi: 10.3389/fphys.2020.00726 32714209 PMC7344166

[50] Tepper BJ , Koelliker Y, Zhao L, et al Variation in the bitter-taste receptor gene *TAS2R38*, and adiposity in a genetically isolated population in Southern Italy. Obesity (Silver Spring). 2008;16:2289–2295. doi: 10.1038/oby.2008.357 18719631

[51] Sausenthaler S , Rzehak P, Wichmann HE, et al Lack of relation between bitter taste receptor TAS2R38 and BMI in adults. Obesity (Silver Spring). 2009;17:937–938; author reply 939. doi: 10.1038/oby.2009.15 19396080

[52] Ozdener MH , Subramaniam S, Sundaresan S, et al CD36- and GPR120-mediated Ca^2+^ signaling in human taste bud cells mediates differential responses to fatty acids and is altered in obese mice. Gastroenterology. 2014;146:995–1005. doi: 10.1053/j.gastro.2014.01.006 24412488 PMC3979457

[53] Owen JA Jr . Physiology of the menstrual cycle. Am J Clin Nutr. 1975;28:333–338. doi: 10.1093/ajcn/28.4.333 1091131

[54] Schmalenberger KM , Tauseef HA, Barone JC, et al How to study the menstrual cycle: practical tools and recommendations. Psychoneuroendocrinology. 2021;123:104895. doi: 10.1016/j.psyneuen.2020.104895 33113391 PMC8363181

[55] Rogan MM , Black KE. Dietary energy intake across the menstrual cycle: a narrative review. Nutr Rev. 2023;81:869–886. doi: 10.1093/nutrit/nuac094 36367830 PMC10251302

[56] Eckel LA. The ovarian hormone estradiol plays a crucial role in the control of food intake in females. Physiol Behav. 2011;104:517–524. doi: 10.1016/j.physbeh.2011.04.014 21530561 PMC3139826

[57] Hirschberg AL. Sex hormones, appetite and eating behaviour in women. Maturitas. 2012;71:248–256. doi: 10.1016/j.maturitas.2011.12.016 22281161

[58] Than TT , Delay ER, Maier ME. Sucrose threshold variation during the menstrual cycle. Physiol Behav. 1994;56:237–239. doi: 10.1016/0031-9384(94)90189-9 7938232

[59] Kuga M , Ikeda M, Suzuki K. Gustatory changes associated with the menstrual cycle. Physiol Behav. 1999;66:317–322. doi: 10.1016/S0031-9384(98)00307-2 10336160

[60] Nagai M , Matsumoto S, Endo J, et al Sweet taste threshold for sucrose inversely correlates with depression symptoms in female college students in the luteal phase. Physiol Behav. 2015;141:92–96. doi: 10.1016/j.physbeh.2015.01.003 25576640

[61] Barbosa DEC , de Souza VR, dos Santos LAS, et al Changes in taste and food intake during the menstrual cycle. J Nutr Food Sci. 2015;5:1. doi: 10.4172/2155-9600.1000383

[62] Byun YH , Koo SJ, Choi JK. Pain threshold & taste threshold variations across the menstrual cycle. J Oral Med Pain. 2001;26:253–260.

[63] Frye CA , Demolar GL. Menstrual cycle and sex differences influence salt preference. Physiol Behav. 1994;55:193–197. doi: 10.1016/0031-9384(94)90031-0 8140168

[64] Olson BR , Forman MR, Lanza E, et al Relation between sodium balance and menstrual cycle symptoms in normal women. Ann Intern Med. 1996;125:564–567. doi: 10.7326/0003-4819-125-7-199610010-00005 8815755

[65] Glanville EV , Kaplan AR. Taste perception and the menstrual cycle. Nature. 1965;205:930–931. doi: 10.1038/205930a0 14281827

[66] Beiguelman B. Taste sensitivity to phenylthiourea and menstruation. Acta Genet Med Gemellol (Roma). 1964;13:197–199. doi: 10.1017/S1120962300015833 14169798

[67] Brug J , Debie S, van Assema P, et al Psychosocial determinants of fruit and vegetable consumption among adults: results of focus group interviews. Food Qual Prefer. 1995;6:99–107. doi: 10.1016/0950-3293(95)98554-V

[68] Spence C. Explaining seasonal patterns of food consumption. Int J Gastron Food Sci. 2021;24:100332. doi: 10.1016/j.ijgfs.2021.100332

[69] Kräuchi K , Wirz-Justice A. The four seasons: food intake frequency in seasonal affective disorder in the course of a year. Psychiatry Res. 1988;25:323–338. doi: 10.1016/0165-1781(88)90102-3 3186862

[70] Van Staveren WA , Deurenberg P, Burema J, et al Seasonal variation in food intake, pattern of physical activity and change in body weight in a group of young adult Dutch women consuming self-selected diets. Int J Obes. 1986;10:133–145. 3013791

[71] Capita R , Alonso-Calleja C. Differences in reported winter and summer dietary intakes in young adults in Spain. Int J Food Sci Nutr. 2005;56:431–443. doi: 10.1080/09637480500407875 16361183

[72] Sturm R , Patel D, Alexander E, et al Seasonal cycles in food purchases and changes in BMI among South Africans participating in a health promotion programme. Public Health Nutr. 2016;19:2838–2843. doi: 10.1017/S1368980016000902 27169872 PMC10270870

[73] Motoki K , Saito T, Nouchi R, et al The paradox of warmth: ambient warm temperature decreases preference for savory foods. Food Qual Prefer. 2018;69:1–9. doi: 10.1016/j.foodqual.2018.04.006

[74] Hull HR , Radley D, Dinger MK, et al The effect of the Thanksgiving holiday on weight gain. Nutr J. 2006;5:29. doi: 10.1186/1475-2891-5-29 17118202 PMC1660573

[75] Seo HS , Buschhüter D, Hummel T. Odor attributes change in relation to the time of the year. Cinnamon odor is more familiar and pleasant during Christmas season than summertime. Appetite. 2009;53:222–225. doi: 10.1016/j.appet.2009.06.011 19576937

[76] Postolache TT , Wehr TA, Doty RL, et al Patients with seasonal affective disorder have lower odor detection thresholds than control subjects. Arch Gen Psychiatry. 2002;59:1119–1122. doi: 10.1001/archpsyc.59.12.1119 12470128

[77] Aso‐Someya N , Kanazawa T, Kudo F. Seasonal variation of taste recognition threshold in young females. FASEB J. 2015;29:LB232. doi: 10.1096/fasebj.29.1_supplement.lb232

[78] de Castro JM. Weekly rhythms of spontaneous nutrient intake and meal pattern of humans. Physiol Behav. 1991;50:729–738. doi: 10.1016/0031-9384(91)90010-L 1775547

